# Assessing Teenage Girls’ Awareness of the Health Risks of Illegal Abortion: A Case Study Protocol in Lusikisiki

**DOI:** 10.3390/ijerph23070931

**Published:** 2026-07-21

**Authors:** Nomzamo Mashumi, Eric Maimela, Nomfuneko Sithole, Thokoe Vincent Makola, Wandile Innocent Dhlamini, Ntiyiso Vinny Khosa

**Affiliations:** 1 School of Public Health, Walter Sisulu University, Mthatha 5099, South Africa; 210082232@mywsu.ac.za (N.M.); emaimela@wsu.ac.za (E.M.); nosithole@wsu.ac.za (N.S.); tmakola@wsu.ac.za (T.V.M.); wdhlamini@wsu.ac.za (W.I.D.); 2 Biostatistics and Analytics Training Services Unit, School of Medicine and Health Sciences, Walter Sisulu University, East London 5200, South Africa; 3 Division of Epidemiology and Biostatistics, School of Medicine and Health Sciences, Walter Sisulu University, East London 5200, South Africa; 4 WSU Institute for Clinical Governance and Healthcare Administration, Faculty of Medicine and Health Sciences, Walter Sisulu University, East London 5200, South Africa; 5 Global Centre for Human Resources for Health Intelligence, Walter Sisulu University, East London 5200, South Africa

**Keywords:** awareness, health risks, illegal abortion, reproductive health services, reproductive health information sources, socio-cultural influences, teenage girls

## Abstract

**Highlights:**

**Public health relevance—How does this work relate to a public health issue?**

- Unsafe and illegal abortion is one of the known contributors to maternal morbidity and mortality, especially among teenagers in resource-poor environments like Lusikisiki, where there might not be equal provision of safe reproductive healthcare services.
- Whether teenage girls know it or not, the dangers associated with illegal abortion have implications for their reproductive health and the likelihood of developing complications such as sepsis and bleeding.

**Public health significance—Why is this work of significance to public health?**

- The proposed qualitative case study aims to assess teenage girls’ awareness or misconception about the dangers associated with unsafe practices, which are crucial in determining public health strategies for improving knowledge on safe abortion practices.
- The anticipated findings of the proposed study could provide useful information in terms of how to improve the quality of reproductive health services, especially in line with national health priorities set by the South African Department of Health.

**Public health implications—What are the key implications or messages for practitioners, policy makers and/or researchers in public health?**

- It is important to improve reproductive education and community awareness campaigns on legal abortions and the laws related to that in order to increase understanding among teenagers.
- It is necessary for policymakers and practitioners to focus more on the provision of reproductive health services to teenagers and to conduct future studies.

**Abstract:**

Illegal abortion remains a significant public health challenge globally, particularly among adolescent girls who often lack adequate reproductive health information, access to healthcare services, and social support. Despite South Africa’s progressive abortion legislation, teenage girls in rural communities like Lusikisiki still face barriers to accessing safe reproductive health services due to socio-cultural stigma, misinformation, and limited youth-friendly services, increasing the risk of unsafe abortion and related health complications. This paper outlines a protocol for a proposed qualitative case study that aims to assess the awareness of teenage girls in Lusikisiki regarding the health risks associated with illegal abortion, explore their sources of information, identify factors influencing their awareness, and propose strategies to reduce unsafe abortion. A qualitative case study design guided by an interpretive paradigm will be used to explore the perceptions and experiences of teenage girls. A purposive sample of 25 participants aged 13–19 years will be recruited from Village Clinic, Bambisana Hospital, and St Elizabeth Regional Hospital. Data will be collected through semi-structured interviews and analysed using thematic analysis supported by NVivo software. The anticipated findings are expected to provide context-specific insights to support improved reproductive health education and adolescent-friendly healthcare services in rural communities.

## 1. Introduction

Illegal abortion is still a major public health and social problem around the world, especially for young girls who are more vulnerable since they do not have easy access to proper information, healthcare, and social support. In the past, South Africa’s abortion laws changed from the strict 1975 Abortion and Sterilisation Act, which only allowed abortion in certain situations, to the more liberal Choice on Termination of Pregnancy Act of 1996, which made abortion legal on request during the first 12 weeks of pregnancy.

Young women will need accurate information about the health hazards associated with illegal abortion to enable informed reproductive health choices and reduce preventable negative outcomes, including maternal morbidity and mortality, particularly in rural and underserved areas [1,2]. Therefore, this protocol outlines the background of the study, including the problem statement, research questions, aims, objectives, justification and significance of the study, and materials and methods. The planned case study will explore the extent to which teenage girls in Lusikisiki, a rural location in the Eastern Cape province of South Africa, are aware of the health hazards associated with illegal abortion. This manuscript presents a protocol for a proposed qualitative case study, guided by an interpretive paradigm, to explore teenage girls’ awareness of the health risks associated with illegal abortion in Lusikisiki, while outlining the study aim, objectives, methodology, ethical considerations and planned data analysis procedures without reporting empirical findings.

### 1.1. Background

In South Africa, abortion law has shifted from restrictive regulation under the 1975 abortion and sterilisation Act to a more progressive legal framework that recognises women’s health and reproductive rights [3,4]. Before 1996, the 1975 Abortion and Sterilisation Act made abortion mostly illegal [4]. It only allowed abortion in very limited situations, such when the mother’s life was in risk, when the foetus had serious problems, or when the pregnancy was the result of rape or incest [3,4,5,6]. This restrictive system contributed to unsafe and illegal abortions, which increased maternal morbidity and mortality, especially among poor and disadvantaged women with limited access to safe reproductive healthcare [7,8,9,10].

The post-apartheid government passed the Choice on Termination of Pregnancy Act in 1996 to recognise the public health issue and fight for women’s rights. This law represents a significant shift, enabling individuals to request an abortion during the first 12 weeks of pregnancy. After that period, abortions can still be performed, but only under specific conditions. This creates a framework that prioritises both choice and safety, reflecting a detailed approach to reproductive rights. This law aimed to make abortion services safe, accessible, and legal, and reduce the risks of unsafe abortions, and support reproductive freedom as part of South Africa’s broader commitment to human rights and gender equality [3,4,5,6].

Unsafe abortion and its consequences are a major global health problem especially in low and middle-income countries. The World Health Organization estimates that approximately 45% of all abortions worldwide are unsafe, with the majority occurring in low- and middle-income countries [11,12]. Adolescents are disproportionately affected by unsafe abortion practices due to limited access to reproductive health information, stigma, socio-cultural barriers, and inadequate youth-friendly health services [13,14,15]. In South Africa, adolescent pregnancy remains a persistent challenge, with Statistics South Africa reporting that thousands of girls aged 10–19 years give birth annually, particularly in rural provinces such as the Eastern Cape [10]. Adolescents in underserved areas are disproportionately affected by unsafe abortion practices due to limited access to reproductive health information, stigma, socio-cultural barriers, and inadequate youth-friendly health services [13,14,15,16]. Consequently, unsafe abortion continues to contribute to maternal morbidity and preventable health complications among adolescent girls [7,9,11,12].

Recent adolescent data further demonstrate why the proposed age range is relevant. In South Africa, public sector data that combine births and legal terminations of pregnancy (TOPs) estimated an adolescent pregnancy rate of 48.9 per 1000 among girls aged 15–19 years in 2024/2025, and 1.2 per 1000 among girls aged 10–14 years [17]. The same analysis notes that South African public data include age-disaggregated TOPs, but exclude miscarriages, private-sector TOPs, not-for-profit services and illegal terminations, meaning that adolescent pregnancy and unsafe abortion burdens are likely underestimated [17]. The Eastern Cape remains relevant to the present study because public-sector data recorded 497 births plus TOPs among girls aged 10–14 years in 2025, and 101 TOPs among this age group in the province [17]. These local figures align with global evidence that about 55% of unintended pregnancies among adolescents aged 15–19 years in low- and middle-income countries end in abortion, often unsafe, while Southern Africa has an estimated termination of pregnancy rate of 30 per 1000 women of reproductive age, with approximately 23% of pregnancies ending in termination [7,18].

Furthermore, unsafe abortion is a major cause of maternal death around the world, accounting for 13% of all maternal fatalities, according to the World Health Organisation [11,12,19,20]. Although many countries have legal laws that permit abortion, unsafe and illegal abortions continue because of social stigma, stringent restrictions, and poor access to healthcare [11,12,15,16,21]. Adolescent girls are more vulnerable because they often have limited access to comprehensive sexual education, and accurate information about the risks of unsafe abortion [13,14,15].

This highlights the continuing need for comprehensive reproductive health education, youth-friendly services, and supportive health policies to reduce unsafe abortion among adolescents [11,13,14,15,22]. The burden of illegal and unsafe abortion remains substantial in Africa because of restrictive abortion regulations, socio-cultural and religious norms, stigma and limited reproductive health infrastructure [7,11,16,21]. Unsafe abortion is thought to be one of the main causes of maternal deaths in Kinshasa, the Democratic Republic of Congo. It also plays a big role in the high rates of maternal morbidity among teenage girls [23].

Teenage girls in many African countries lack enough knowledge about the risks of illegal abortion, and this is made worse by problems like poverty, gender inequality, and poor sex education [13,14,15,23]. These things make it clear that it is very important to raise awareness about reproductive health among African teenagers.

Since 1996, abortion has been legal in South Africa under the Choice on Termination of Pregnancy Act. Women can terminate the pregnancy on request up to 12 weeks into their pregnancy and under specific conditions after that [4,5,6]. Despite the availability of reproductive health services and a progressive legal system, illegal abortion and the health risks remain important public health, especially for vulnerable groups like teenage girls in rural areas [8,14,15,16].

Ongoing dangerous behaviours are caused by things like shame, not knowing existing legal rights, and not being able to get private treatments. Evidence shows that unsafe abortion is associated with maternal morbidity and preventable complications, particularly in settings where access to safe and illegal abortion care remains limited [7,9,11,12]. In rural South African contexts, including areas such as Eastern Cape province, reproductive health may be constrained by poverty, health system barriers and socio-cultural norms [8,14,15,16].

While some research has been conducted on teenage reproductive health in South Africa, few studies have focused specifically on teenage girls’ awareness of the health risks of illegal abortion in Lusikisiki or similar rural Eastern Cape contexts. This gap signals a need for localised data to inform community-specific health education strategies and reproductive health services that address knowledge deficits, reduce unsafe abortion rates, and improve adolescent health outcomes.

### 1.2. Problem Statement

The problem to be explored is the insufficient awareness among teenage girls in Lusikisiki regarding the health risks associated with illegal abortion. This is a major public health concern because unsafe abortion remains an important contributor to maternal morbidity and mortality around the world, and it affects adolescent girls in rural and underserved areas more than other groups particularly in low-and middle countries [11,12]. The World Health Organisation estimates that roughly 25 million unsafe abortions occur annually worldwide, almost all in low and middle-income countries, and adolescents are disproportionately affected, with higher complication rates reported in rural and underserved areas [11,12]. Therefore, to reduce preventable deaths and improve reproductive health outcomes, it is important to fill in the gaps in knowledge about unsafe abortion [24].

This is in line with Sustainable Development Goal (SDG) 3 and Africa’s Agenda 2063, both of which promote health and well-being for all at all ages [25]. Many adolescent girls in communities like Lusikisiki still remain unaware of the hazards of illegal abortion and the availability of safe procedures [9,12], even though abortion is legal in South Africa. This unawareness keeps risky practices going. As a result, these adolescent girls suffer from acute clinical complications such as severe haemorrhage, sepsis, septic shock, uterine perforation, genital tract and abdominal injuries, and incomplete abortion/retained products of conception requiring emergency care [9,12].

While the immediate consequences of unsafe abortion are often manifested through severe physical and reproductive health complications, the effects frequently extend beyond physical health to include significant psychological, emotional, and social challenges.

Previous studies have highlighted the need for surgical interventions such as uterine evacuation (manual vacuum aspiration or dilation and curettage), laparotomy for bowel or uterine injury, and, in severe cases, hysterectomy to control life-threatening bleeding or infection [9,11,12,23]. Fertility-related sequelae such as intrauterine adhesions (Asherman’s syndrome) after repeated curettage or infection, and secondary infertility risk when severe pelvic inflammatory disease occurs, recognising that infertility is not an inevitable outcome and depends on the severity and management of complications [11,12].

Beyond these physical and reproductive health consequences, illegal abortion can also have profound psychological, emotional and social implications for adolescent girls [14,15,16,23]. The effects often extend beyond immediate medical complications and may influence mental health, educational attainment, family relationships and broader social wellbeing.

Furthermore, previous studies highlight longer-term health effects such as chronic pelvic pain, anaemia from blood loss, and increased risk of future adverse pregnancy outcomes after severe sepsis or uterine injury [9,11,12]. Whilst other studies underline the psychosocial and educational impacts, such as trauma, anxiety or depression, stigma, relationship conflict, and school interruption or dropout, especially where confidentiality and access to supportive care are limited [14,15,16]. Abortion-related deaths and severe complications may persist where access to safe services and timely post-abortion care is limited [9,11,12,22]; adolescents face additional risk due to delays and secrecy.

This gap between available services and what adolescent girls know and can access makes it harder to design targeted interventions that meet their specific needs. This study’s goal is to fill this gap by looking at how much teenage girls in Lusikisiki know about the health risks of illegal abortion. It will use successful methods from other studies that stress community-based education, health services that are friendly to teens, and communication that is sensitive to cultural differences.

### 1.3. Justification of the Study and Significance

This manuscript presents a study that is justified by the continued public health concern posed by illegal abortion among adolescent girls in rural South Africa. Despite the legal availability of abortion services under the Choice on Termination of Pregnancy Act, evidence suggests that many adolescents continue to utilize unsafe abortion methods due to limited awareness, stigma, socio-cultural influences and barriers to accessing reproductive healthcare services. Rural communities such as Lusikisiki face additional challenges related to poverty, healthcare accessibility and reproductive health education.

Lusikisiki was selected because it represents a predominantly rural setting within the Eastern Cape Province, where adolescent pregnancy rates remain high and access to reproductive health information may be limited. Understanding adolescent awareness of the health risks associated with illegal abortion in this context is important for informing targeted health education programmes, policy interventions and adolescent reproductive health services. Furthermore, increasing attention to adolescent sexual and reproductive health in South Africa highlights the need for context-specific evidence to support effective prevention strategies and improve health outcomes among vulnerable populations.

### 1.4. Research Objectives

#### 1.4.1. Overall Objective

To assess the awareness of teenage girls in Lusikisiki about the health risks of illegal abortion.

#### 1.4.2. Specific Objectives

To explore the sources used by teenage girls in Lusikisiki to obtain information about abortion and its associated risks.

To identify and describe factors influencing awareness of illegal abortion among teenage girls in Lusikisiki.

To explore the strategies to mitigate illegal abortion on teenage girls’ health and well-being in Lusikisiki.

## 2. Materials and Methods

### 2.1. Study Design

This protocol describes a proposed qualitative case study guided by an interpretive paradigm to explore teenage girls’ awareness of the health consequences of illegal abortion in Lusikisiki. The case study design is appropriate because it enables an in-depth examination of a bounded rural setting within its real-life social, cultural and healthcare context [26,27]. It will allow the researcher to generate rich, context-specific data on adolescents’ awareness, sources of information and factors shaping their understanding of illegal abortion risks.

### 2.2. Study Setting

The study will be conducted in Lusikisiki, a predominantly rural town in the Ingquza Hill Local Municipality of the OR Tambo District, Eastern Cape. This study setting was selected due to the lack of local research on teenage girls’ awareness of the health risks associated with illegal abortion. The area is characterised by high levels of poverty, unemployment, and limited access to healthcare services, with healthcare mainly provided through clinics, community health centres, and referral hospitals.

### 2.3. Population

The population for this proposed study comprises teenage girls aged 13 to 19 years from Lusikisiki Village clinic, Bambisana hospital, and St Elizabeth Regional Hospital in the Eastern Cape. This age range encompasses mid- to late adolescence, a critical period for sexual and reproductive health education and decision-making. The proposed study focuses on this group as they are particularly vulnerable to unsafe abortion practices due to limited knowledge and socio-cultural constraints [13,14,15,16]. The population in Lusikisiki is predominantly isiXhosa-speaking.

### 2.4. Sampling Method

A purposive sample of teenage girls aged 13–19 years who are accessing reproductive health services at selected healthcare facilities in Lusikisiki, including Lusikisiki Village Clinic, Bambisana Hospital, and St Elizabeth Regional Hospital, will be invited to participate. The study will primarily recruit participants from selected healthcare facilities because these settings provide access to adolescents seeking reproductive health services, including those who may have prior knowledge or indirect experiences related to abortion. However, illegal abortion often occurs outside formal healthcare settings. To address this, snowball sampling will be used to recruit adolescents who are not currently accessing healthcare services but have relevant experiences or knowledge. This combined approach enables the inclusion of both facility-based participants and harder-to-reach individuals, thereby strengthening the study’s ability to capture a broader range of perspectives on illegal abortion.

### 2.5. Inclusion and Exclusion Criteria

Inclusion Criteria include:

- Teenage girls aged 13–19 years;
- Able to communicate in isiXhosa or English;
- Willing to participate in the study;
- Provided informed consent or assent, with parental/guardian consent where applicable.

### 2.6. Study Procedure

Ethical approval will first be sought from the Faculty of Medicine and Health Sciences at Walter Sisulu University to obtain a Human Ethics Clearance Certificate. After ethical clearance has been granted, permission to conduct the study in the OR Tambo District will be requested from the Eastern Cape Department of Health. Following approval from the Eastern Cape Department of Health, permission will then be requested from the OR Tambo District Department of Health and the relevant facility gatekeepers. Once all approvals have been obtained, the researcher will finalise the study documents, including the approved study protocol, informed consent and assent forms, parental or guardian consent forms, and the participant interview guide. Study materials will be translated into isiXhosa to ensure comprehension.

### 2.7. Recruitment

The proposed study will employ purposive sampling to recruit 25 teenage girls aged 13–19 years from selected healthcare facilities in Lusikisiki. Nurses and facility managers will be consulted to assist with coordinating recruitment and identifying suitable times for interviews so that data collection does not interfere with routine healthcare services. They may also assist in directing the researcher to potential participants who meet the inclusion criteria; however, participation will remain voluntary, and the researcher will be responsible for explaining the study and obtaining consent or assent.

Eligible participants will be approached according to the study inclusion criteria. The purpose of the study, study procedures, potential risks, benefits, confidentiality measures, voluntary participation and the right to withdraw without penalty will be explained to each participant. Written informed consent will be obtained from participants aged 18–19 years. For participants younger than 18 years, parental or guardian consent and participant assent will be obtained before participation.

### 2.8. Data Collection Preparation

The data collection tools will be piloted prior to the main data collection process using three participants who meet the study’s inclusion criteria, but who will not be included in the final sample. This piloting process will assess the clarity, relevance, and comprehensibility of the interview questions, as well as identify any ambiguities and estimate the duration of the interviews. Feedback obtained from this process will be used to refine and improve the interview guide before the commencement of the main study.

### 2.9. Data Collection

Data will then be collected through semi-structured interviews conducted in isiXhosa by the researcher using a pre-tested interview guide. The interviews will explore participants’ awareness of illegal abortion risks, sources of information and factors influencing their awareness. Interviews will be audio-recorded with participants’ permission and conducted in private, neutral locations to ensure confidentiality and participant comfort. The researcher intends to collect data over a two-month period. During this time, the researcher will spend approximately 2–3 weeks at each selected healthcare facility in Lusikisiki to recruit eligible participants and conduct interviews. Each interview will last approximately 30–45 min. Data collection will adhere strictly to ethical principles, including confidentiality, anonymity, voluntary participation and respect for participants’ dignity. Participants who experience distress during the interview process will be referred to appropriate counselling services.

### 2.10. Retention Data

The researcher will refer to the number of participants agreed to participate and successfully complete the interview process during the data collection period. The case study design will focus on single interviews. Therefore, participants will not be followed over time. However, retention will be measured by the proportion of recruited participants who complete the interview.

The researcher will take note of the total number of eligible participants approached, the number of participants who consent to participate, the number of participants who complete the interview, and the number of participants who withdraw before or during the interview. The data will help the researcher to estimate the average number of patients presenting to the selected healthcare facilities with complications associated with illegal abortion during the study period.

### 2.11. Data Management

All data collected during the study will be managed in line with Walter Sisulu University research policy, the National Health Research Ethics Council (NHREC) guidelines, and international ethical standards, with emphasis on confidentiality, accuracy, and integrity. Completed consent forms and data entry will be reviewed daily by the principal investigator to check for accuracy, completeness, and consistency. Hard-copy documents will be stored in a locked cabinet accessible only to the researcher and if necessary, the supervisor, while electronic data will be captured, transferred, coded, and stored on a password-protected computer. To further safeguard participant information, identifying details will not be included in the dataset; instead, unique identification codes will be assigned to each participant.

#### Access and Control

Access to study data will be strictly limited to the principal investigator and academic supervisors. After data analysis and dissemination of findings, the anonymised datasets will be archived securely for a minimum of five years, in line with Walter Sisulu University policy. Thereafter, all hard-copy data will be shredded, and electronic files permanently deleted from storage devices to protect participant confidentiality.

### 2.12. Data Collection Instrument

Data will be collected through semi-structured interviews conducted in isiXhosa using a pre-tested interview guide consisting of seven sections covering participants’ demographic characteristics, awareness of abortion and its health risks, sources of information, socio-cultural influences, barriers to accessing reproductive health information and services, perceived consequences of illegal abortion, and additional comments. Interviews will be audio-recorded with participants’ consent, supported by field notes, and guided by probing questions to obtain rich and detailed responses.

### 2.13. Data Entry and Analysis

Thematic analysis will be used to analyse the qualitative data, involving systematic coding and identification of key themes and patterns [28].

The researcher will conduct thematic analysis following Ref. [28] six-step framework, namely, familiarisation through repeated reading of transcripts while noting initial patterns; generating initial codes systematically across the dataset to identify features of interes, searching for themes by collating codes into potential themes and subthemes [6]. The researcher will utilise qualitative data analysis software, such as NVivo 14 (Lumivero), where possible, to systematically organise and manage the coded data [28]. The researcher will utilise qualitative data analysis software, such as NVivo 14 (Lumivero), where possible, to systematically organise and manage the coded data.

### 2.14. Dissemination of Information

The anticipated findings of the study will be disseminated through a final report submitted to Walter Sisulu University and the Eastern Cape Department of Health, including the participating healthcare facilities in Lusikisiki. The results may also be shared through conference presentations and publication in peer-reviewed journals. To provide feedback to the study setting, a plain-language summary of the findings will be made available through the participating clinics and hospitals for participants and facility stakeholders who express an interest in the study outcomes.

### 2.15. Ethical Considerations

Ethical approval for the proposed study was obtained from the Walter Sisulu University Health Sciences Research Ethics Committee, as well as the Eastern Cape Department of Health, OR Tambo District department of health and relevant gatekeepers. All study procedures will be conducted in accordance with approved ethical guidelines for research involving human participants.

## 3. Discussion and Conclusions

This protocol aims to examine teenage girls’ awareness of the health risks associated with illegal abortion in Lusikisiki, a rural area in the Eastern Cape, thereby contributing to broader efforts to address adolescent sexual and reproductive health inequalities [13,14,15,17,25]. By focusing on a marginalised rural setting, the study aligns with global priorities on health equity and the reduction in sexual and reproductive health disparities among adolescents, while highlighting how geographical, socio-economic, and health-system constraints shape adolescents’ knowledge and perceptions of unsafe abortion [8,13,14,15,16].

The interpretation of anticipated findings will be guided by the Socio-Ecological Model and Feminist Political Ecology, providing a multi-level understanding of how individual, social, and structural factors influence awareness and reproductive autonomy. A key strength of this qualitative case study lies in its ability to generate in-depth, context-specific insights through semi-structured interviews, capturing adolescents lived experiences and perspectives that are often underrepresented in research [26,27].

The anticipated findings are expected to inform culturally sensitive reproductive health education, strengthen youth-friendly service delivery, and support community-based interventions aimed at reducing unsafe abortion and improving adolescent health outcomes. Ultimately, this study will contribute to evidence-based policy and practice, promoting equitable access to sexual and reproductive healthcare and reducing preventable maternal morbidity among adolescents in similar low-resource settings [12,29].

## 4. Limitations and Mitigation Strategies

### 4.1. Limitations

This study may face several limitations. Social desirability bias is possible, as participants may underreport or alter responses due to stigma surrounding abortion. The focus on a specific rural setting may limit the generalisability of findings to other contexts, and the sensitive nature of the topic may reduce willingness to participate, affecting sample size and diversity. Additional challenges include accessibility barriers and potential language or comprehension difficulties, which may influence the depth and accuracy of responses. Consequently, the findings may not be fully transferable to all adolescent populations in South Africa.

### 4.2. Mitigation Strategies

To address these limitations, the study will ensure a confidential, non-judgmental interview environment and employ culturally sensitive interviewers to encourage open discussion. Flexible scheduling, private interview settings, purposive and snowball sampling will be used to improve participation and reach adolescents who may not access healthcare services. Interviews will be conducted in participants’ preferred languages to minimise language barriers, and detailed contextual descriptions will be provided to help readers assess the relevance of the findings to other rural or underserved settings.

## Data Availability

The anticipated findings of the research project will be available in the form of a dissertation and a journal article. The anticipated findings will also be available to Walter Sisulu University, the faculty of medicine and health sciences, and to other researchers via publication and conferences. Further inquiries can be directed to the corresponding author.
